# Iconography of abnormal non-neuronal cells in pediatric focal cortical dysplasia type IIb and tuberous sclerosis complex

**DOI:** 10.3389/fncel.2024.1486315

**Published:** 2025-01-06

**Authors:** Joyce Zhang, Deneen Argueta, Xiaoping Tong, Harry V. Vinters, Gary W. Mathern, Carlos Cepeda

**Affiliations:** ^1^IDDRC, Jane and Terry Semel Institute for Neuroscience and Human Behavior, University of California - Los Angeles, Los Angeles, CA, United States; ^2^Department of Anatomy and Physiology, Shanghai Jiao Tong University School of Medicine, Shanghai, China; ^3^Department of Pathology and Laboratory Medicine, University of California - Los Angeles, Los Angeles, CA, United States; ^4^Department of Neurosurgery, David Geffen School of Medicine, University of California - Los Angeles, Los Angeles, CA, United States

**Keywords:** focal cortical dysplasia, tuberous sclerosis complex, balloon cells, electrophysiology, pediatric epilepsy

## Abstract

Once believed to be the culprits of epileptogenic activity, the functional properties of balloon/giant cells (BC/GC), commonly found in some malformations of cortical development including focal cortical dysplasia type IIb (FCDIIb) and tuberous sclerosis complex (TSC), are beginning to be unraveled. These abnormal cells emerge during early brain development as a result of a hyperactive mTOR pathway and may express both neuronal and glial markers. A paradigm shift occurred when our group demonstrated that BC/GC in pediatric cases of FCDIIb and TSC are unable to generate action potentials and lack synaptic inputs. Hence, their role in epileptogenesis remained obscure. In this review, we provide a detailed characterization of abnormal non-neuronal cells including BC/GC, intermediate cells, and dysmorphic/reactive astrocytes found in FCDIIb and TSC cases, with special emphasis on electrophysiological and morphological assessments. Regardless of pathology, the electrophysiological properties of abnormal cells appear more glial-like, while others appear more neuronal-like. Their morphology also differs in terms of somatic size, shape, and dendritic elaboration. A common feature of these types of non-neuronal cells is their inability to generate action potentials. Thus, despite their distinct properties and etiologies, they share a common functional feature. We hypothesize that, although the exact role of abnormal non-neuronal cells in FCDIIb and TSC remains mysterious, it can be suggested that cells displaying more glial-like properties function in a similar way as astrocytes do, i.e., to buffer K^+^ ions and neurotransmitters, while those with more neuronal properties, may represent a metabolic burden due to high energy demands but inability to receive or transmit electric signals. In addition, due to the heterogeneity of these cells, a new classification scheme based on morphological, electrophysiological, and gene/protein expression in FCDIIb and TSC cases seems warranted.

## 1 Introduction and pathological findings in FCDIIb and TSC

Malformations of cortical development (MCD) comprise a wide range of conditions arising from anomalies in cell differentiation, migration, and proliferation within the cerebral cortex (Barkovich et al., 2012). Although the exact prevalence of MCD remains uncertain, studies estimate their involvement in up to 40% of cases with pharmaco-resistant childhood epilepsies (Barkovich et al., 2012; Guerrini and Dobyns, 2014; Represa, 2019). In such instances, elective surgical resections not only offer a means to mitigate epileptic episodes, but also present an opportunity to explore the pathophysiology of MCD (Blumcke et al., 2017; Juric-Sekhar and Hevner, 2019; Blumcke, 2024).

A common type of MCD is Focal Cortical Dysplasia (FCD), characterized by Taylor et al. while performing microscopic analysis of lobectomy specimens from epileptic patients (Taylor et al., 1971). The study described localized disruptions in cortical laminae and the presence of large, bizarre neurons scattered throughout all but the first cortical layer. In most cases, “grotesque” cells, probably of glial origin, were also present in the depths of the affected cortex and in the subjacent white matter. These “grotesque cells” are now known as balloon cells (BC) due to their peculiar shape. The International League Against Epilepsy (ILAE) consensus classification of FCD distinguishes the pathology into three main classes: FCDI, FCDII, and FCDIII. FCDII is characterized by pronounced cortical dyslamination, the presence of dysmorphic, cytomegalic neurons (FCDIIa), and all of the above plus BC (FCD IIb) (Blumcke et al., 2011; Najm et al., 2022). On Magnetic Resonance Imaging (MRI), there is cortical thickening, aberrant sulcal and gyral patterns, subcortical white matter hyperintensity, and the occurrence of the transmantle sign, which is a funnel-shaped high T2/FLAIR correlated with the presence of abundant BC (Blumcke et al., 2011; Kimura et al., 2019). The transmantle sign is associated with abnormal radial glial progenitor cells, which normally create a framework for neuronal migration from the periventricular germinal matrix to the cortex (Castillo, 2002). Topographic characterization showed that BC are primarily clustered in the white matter and scatter diffusely into the gray-white matter junction, in line with MRI findings (Rossini et al., 2017).

Interestingly, Taylor and associates noticed that FCD with BC displayed histological similarities with tubers isolated from patients with Tuberous Sclerosis Complex (TSC, formerly known as Bourneville disease or epiloia), another rare MCD. Its clinical presentation includes a classic triad of symptoms; epilepsy (particularly infantile spasms), intellectual disability, and facial angiofibromas (Northrup et al., 2021). Other manifestations include cortical tubers, subependymal nodules, subependymal giant cell astrocytomas (SEGA), cardiac rhabdomyomas, renal angiomyolipomas, retinal hamartomas, pulmonary lymphangioleiomyomatosis, and autism spectrum disorder. TSC results from mutations in TSC1 and TSC2 genes, which code for hamartin and tuberin, respectively. TSC1 and TSC2 inhibit the mechanistic target of rapamycin (mTOR) pathway, which is a major contributor to enhanced protein synthesis and cell growth (Kwiatkowski and Manning, 2005; Liu and Sabatini, 2020; Panwar et al., 2023). Notably, classic features of FCD, such as blurred boundaries of gray and white matter, cortical thickening, and the radial band sign, can also be observed on MRI in TSC cases (Muhlebner et al., 2019). At present, it is not clear whether the radial band in TSC and the transmantle sign in FCDIIb are manifestations of the same pathology or if they are separate conditions (Matsuo et al., 2022). Importantly, in cortical tubers enlarged cells similar to the BC also abound and they have been named “giant” cells (GC). Based on similarities between histopathologic cortical abnormalities observed in FCDIIb and TSC, it has been suggested that FCD with BC represents a forme fruste or phenotypic variant of TSC, limited to selected focal regions of brain tissue (Vinters et al., 1993; Jozwiak et al., 2006). While both share morphological features and protein expression, BC and GC differ in cortical localization. BC in FCDIIb are more concentrated in deep cortical layers and white matter, while GC in TSC are more scattered throughout the tuber and they also are present in perituberal areas (Marcotte et al., 2012). Further, a gross cell count of BC vs. GC from resected tissue showed that GC were more numerous (Cepeda et al., 2012), potentially because GC concentrate within the tubers of TSC lesions and are less common in the surrounding areas (Abdijadid et al., 2015).

A 2020 study used web-based deep learning to delineate unique histopathological features of TSC vs. FCDIIb cortical tissue, two pathological entities hard to differentiate based on Hematoxylin & Eosin (H&E) staining. Although some features appeared unique to TSC samples (e.g., the matrix reaction was fibrillar and strand-like vs. diffuse and granular in FCDIIb, or larger nuclei of astrocytes with uncondensed chromatin vs. smaller nuclei and more condensed chromatin in FCDIIb), BC/GC themselves were not critical to distinguish both pathologies (Kubach et al., 2020). Apparently, the only notable difference between these cells was the presence of a “halo” effect present in GC from TSC but less prevalent in BC from FCDIIb cases (Kubach et al., 2020). The “halo” effect, visible as a ring of white background in H&E staining, is probably caused by altered synaptogenesis in BC/GC (Yamanouchi et al., 1997).

**Figure 1:**
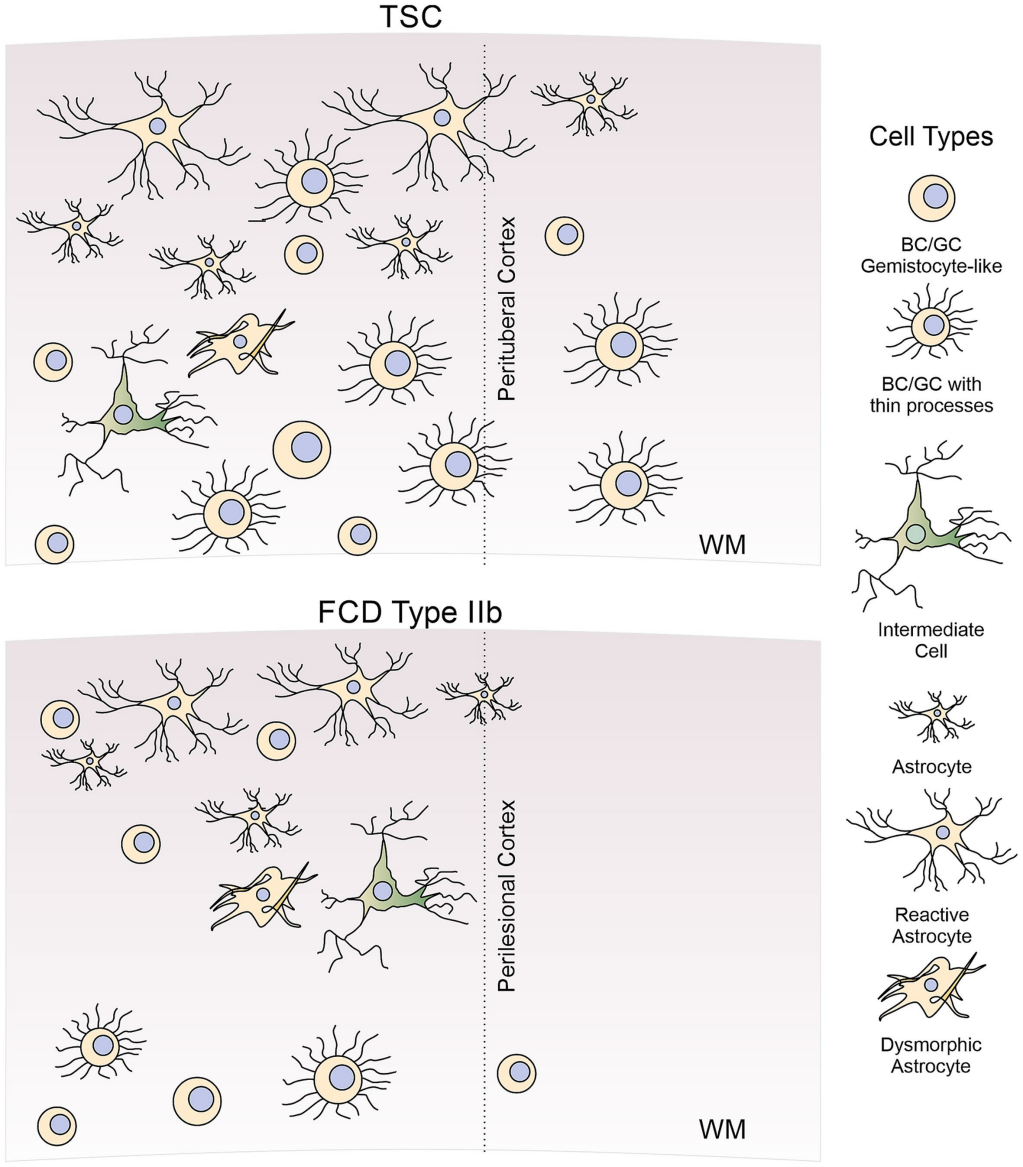
Diagram of abnormal non-neuronal cells found in FCDIIb and TSC cases. In this review, we discuss morphological, molecular, and electrophysiological properties of abnormal non-neuronal cells observed in cortical tissue from FCDIIb and TSC cases. These include BC/GC (both gemistocytic-like and BC/GC with thin processes), intermediate or hybrid cells, reactive, and dysmorphic astrocytes. For comparison, normal astrocytes are also illustrated. Not included in this review are other types of abnormal cells (e.g., dysmorphic cytomegalic neurons), oligodendrocytes or microglia.

The presence of BC/GC in FCDIIb and TSC has baffled histopathologists as they have defied classification due to their ill-defined, glio-neuronal nature. With the advent of imaging techniques allowing visualization of individual cells in *ex vivo* brain slices, e.g., infrared differential interference contrast (IR-DIC) microscopy, a functional characterization of these enigmatic cells is within reach. The present review aims to provide a more detailed characterization of abnormal non-neuronal cells, e.g., BC/GC, intermediate cells, and reactive/dysmorphic astrocytes (Figure 1), in FCDIIb and TSC, with particular emphasis on our own studies in a large cohort of pediatric patients undergoing surgery for the treatment of pharmaco-resistant epilepsy.

## 2 Some words regarding cell nomenclature and definition of BC/GC

BC and GC are named for their substantial size and mostly spherical morphology, hypothesized to result from mutations affecting the mTOR pathway (Miyata et al., 2004; Blumcke et al., 2011). They are similar to gemistocytic astrocytes and to cells found in SEGA. Traditionally, the term “balloon” has been applied to the bizarre cells occurring in FCDIIb, while the term “giant” has been reserved to the enlarged cells observed in TSC. According to the ILAE classification of FCDs, BC are characterized by a large cell body, opalescent glassy eosinophilic cytoplasm lacking Nissl substance, and the frequent occurrence of multiple nuclei (Blumcke et al., 2011). On the other hand, the term “giant” in TSC refers to a cell type that shares a large soma, as well as cytoplasmic and nuclear characteristics also encountered in BC of FCDIIb. However, the term “giant” is fraught with confusion due to its lack of specificity. There are other cells in cortical tubers that also are very large, e.g., cytomegalic neurons, but are unlike “balloon” cells. But besides their occurrence in different pathologies, is there a good reason to divide the abnormally enlarged, non-neuronal cells into “balloon” and “giant”? Probably not. In fact, some of the current literature has used BC and GC interchangeably, regardless of the associated pathology (Vinters et al., 1993; Fauser et al., 2004; Yasin et al., 2010; Kubach et al., 2020; Arceneaux et al., 2024; Liu et al., 2024). In this review we use the general term BC/GC to identify this specific type of cell regardless of underlying pathology, generally BC when applied to FCDIIb, and GC when applied to TSC cases. We reserve the term “non-neuronal” cells to encompass any cell type unable to generating action potentials including BC, GC, intermediate cells, dysmorphic astrocytes, as well as the normal and reactive astrocytes. Not included in this review are oligodendrocytes and microglia, which also are affected in TSC and FCDIIb (Boer et al., 2006; Gruber et al., 2021).

## 3 A brief history of BC/GC in TSC and FCDIIb

The first detailed microscopic description of FCD, including the identification of “grotesque” cells similar to those reported in TSC, occurred relatively recently (Taylor et al., 1971). In contrast, histological observations of large, “atypical” cells in cortical tubers started in the early 20th century. It was probably G. B. Pellizzi the first neuropathologist who, in 1901, described the dysplastic nature of the tubers and the existence of heterotopias in the brains of TSC cases (Pellizzi, 1901). Abnormal cells were found inside the tubers and they were variably referred to as “atypical,” “bizarre,” “giant,” “monstrous.” From the outset, the identity of these cells was a puzzle, due to their glio-neuronal aspect. Hallervorden and Krücke, in a paper published in 1956 [cited in (Arseni et al., 1972)], maintained that the large cells in TSC represent a heterogeneous cellular group formed by both nerve and glial cells. They also mentioned that these cells are malformed, undifferentiated neurons, “indifferent” forms of transitions from glial cells to neurons. Ultrastructural studies also were published in the late 1960s and 1970s (Gruner, 1969; Ribadeau Dumas et al., 1973). Of particular interest is the case of a stillborn infant (31st week gestation) who presumably died due to a rhabdomyoma of the heart. Notably, the “atypical” cells in the cortical tuber showed ultrastructural features of reactive astrocytes adorned with innumerable microvilli-like projections on their surface and junctional complexes (Probst and Ohnacker, 1977). In addition, some features of these “atypical” cells resembled those of reactive astrocytes and gemistocytes [round to oval astrocytes with abundant, glassy, eosinophilic cytoplasm and an eccentric nucleus (Tihan et al., 2006)]. Probst and Ohnacker suggested that the “atypical” cells in this case were the manifestation of aberrant differentiation of progenitor cells. Apparent discrepancies regarding the glio-neuronal ambiguity of the “monstrous” cells described by other authors, they remarked, could be due to the localization of the tuber sample and different stage of differentiation.

The Golgi method provided invaluable information on the fine morphology of brain cells in both normal and pathological conditions, including TSC, other cortical malformations, and subcortical heterotopias (Ferrer, 2024). In 1984, three landmark papers analyzed the fine morphology of neurons and glia in cortical tubers using the Golgi technique (Ferrer et al., 1984; Huttenlocher and Heydemann, 1984; Machado-Salas, 1984). Huttenlocher and Heydemann described two principal cell types composed of astroglia and “stellate” neurons with varicose dendrites and few dendritic spines, many of those cells lacked identifiable axons. Glial cells were prominent in subpial regions and in deeper zones (Huttenlocher and Heydemann, 1984). Ferrer et al. also noticed a large number of “stellate” cells in the intermediate and deep regions of the tuber. Their main finding was aberrant cellular orientation and impaired neuronal distribution, leading them to postulate a disorder of cell migration and neuronal organization (Ferrer et al., 1984). Machado-Salas identified two main cell populations that included astrocytes, mostly of the fibrillary type, and large pyramidal neurons with misoriented apical dendrites. In addition, a small number of “bizarre” cells of questionable nature, probably pyramidal-like cells with progressive loss of pyramidal contour were observed (Machado-Salas, 1984). He concluded that two types of giant cells coexist in TSC, one type clearly displays nerve cell features, while the other displays the typical morphology of astrocytes.

Modern studies using the Golgi technique have concentrated more on the dendritic and spine morphology of normal and dysmorphic neurons in different types of FCD. Dendritic and spine abnormalities were more evident in normal and dysmorphic neurons of FCDIIb than in FCDIIa (Rossini et al., 2021). Abnormalities were manifested by reduced dendritic fields, spine loss, distortions in spine morphology, and the presence in some cells of numerous dendritic varicosities. Interestingly, in some dysmorphic neurons, the authors observed the presence of numerous short filopodia-like protrusions emerging from the soma. One may wonder if those protrusions are similar to the microvilli reported in “atypical” cells of cortical tubers by Probst and Ohnacker (op. cit.). Another important ultrastructural study of a TSC case and a subependymal tumor found, in both cases, GC with astrocytic characteristics (Trombley and Mirra, 1981). Notably, the authors also reported that, in addition to numerous glial-glial contacts, rare neuroglial junctions were encountered in the cortical tuber case, suggesting aberrant synapse formation. This unprecedented observation, they suggested, may correlate with the existence of transient axo-glia junctions, including synapses, in the developing nervous system. These contacts may promote synaptogenesis by releasing GABA from the glial processes into the neuronal milieu (Wolff et al., 1979). Other electron microscopy studies demonstrated increased intermediate filaments in BC/GC from FCDIIb and TSC cases, along with numerous mitochondria, which were centrally located, without neurosecretory granules, and of normal architecture (Yasin et al., 2010). It should be noted that mTOR is functional in mitophagy inhibition (Frauscher et al., 2017), so increased mitochondrial numbers support the hypothesis that mTOR is overactive in BC/GC.

Pioneer histopathological and IHC studies by Vinters and his group at the University of California, Los Angeles (UCLA) characterized cortical tissue associated with infantile spasms. Those studies concluded that, as already mentioned, cases of severe FCD had similarities to cerebral changes described in TSC, including the presence of blurred gray-white matter junction containing bizarre gemistocytic BC (Vinters et al., 1993). BC in FCDIIb are similar to GC observed in cortical tubers and show both neuronal and astrocytic epitopes, indicating the local proliferation of multipotential neuroectodermal cells (De Rosa et al., 1992). A further characterization of BC demonstrated their location in FCD lesions and presence of GFAP and the cytoskeletal marker tau (Vinters et al., 1999). A revealing study by the same group used autopsy material from a 20-week-old fetus with TSC, which demonstrated three tubers populated with “gemistocyte-like BC,” along with scattered BC throughout the subcortical white matter. These cells were noted to be positive for both GFAP and vimentin (Park et al., 1997). In another landmark report, the development of TSC lesions in fetal brain tissue from 19 gestational weeks to term, it was found that subcortical lesions forming around the germinative zones are the first alterations detected already at 19 weeks of gestation. These lesions are characterized first by the presence of dysmorphic astrocytes and GC. The data suggested that cortical tuber formation is a long process that initiates with the presence of dysmorphic astrocytes and GC, while the appearance of dysmorphic neurons occurs by the end of gestation (Gelot and Represa, 2020). Overall, these studies suggest BC/GC have a mixed phenotype and originate very early during brain development.

## 4 Immunohistological, Western blot, and mRNA expression studies provide insights into the developmental origin and identity of BC/GC

The normal process of cortical development guides the understanding of BC/GC identity. After formation of the neural tube, radial glial cells tightly anchored to each other divide to form a ventricular zone. These radial glial cells are not only the precursors to neurons and glia, but also use their processes to guide proliferating cells away from the ventricles, eventually forming the subventricular zone and the cortex (Zarzor et al., 2023).

Immunohistochemical (IHC) studies have stressed the remarkable similarities of large cells in FCDIIb and TSC, including the expression of neuronal and astrocytic markers such as microtubule-associated protein 2 (MAP2) and glial fibrillary acidic protein (GFAP) (Jozwiak et al., 2005; Ying et al., 2005). Since then, many other specific markers have been added to the list and have aided in determining the origin and identity of BC/GC.

### 4.1 Immature neuronal and glial cell markers

The presence of immature neuronal and glial cell markers in BC/GC (Table 1), along with their localization in the gray/white matter junction, suggest BC/GC may represent cells in a pre-differentiation state (Englund et al., 2005; Rossini et al., 2017). Hence, from the outset it has been difficult to determine if they are more neuronal or glial in origin. The demonstration that radial glia are capable of generating astrocytes as well as neurons during cortical development (Malatesta et al., 2000; Alvarez-Buylla et al., 2001; Noctor et al., 2001) provided important clues about the origin of BC/GC. It seemed possible that BC/GC were originally radial glia that, under unspecified circumstances, had an arrest in development, preventing them from reaching their final morphological and functional fate (Cepeda et al., 2006; Lamparello et al., 2007). Multiple cell markers suggest BC/GC may be arrested at the G1 stage of the cell cycle (Table 1). In BC, low levels of cyclin D and cyclin E suggested cells did not advance to S, as these cyclins facilitate progression through G1 (Thom et al., 2005; Schick et al., 2007b; DeBerardinis et al., 2008). MCM2, a G1 protein necessary for S stage initiation, was expressed in BC/GC, but exposure to stem cell mitogens in BC showed no proliferation (Yasin et al., 2010). These authors also succeeded at isolating, in culture, an undifferentiated population of BC from surgical resections of FCD and cortical tubers, and demonstrated that *β*1-integrin, a protein that participates in neuronal differentiation, labels a sub-population of BC with a stem cell phenotype (Yasin et al., 2010). Ki-67, another cell proliferation marker, was shown to be increased in GC but not expressed in BC (Crino et al., 1996; Munakata et al., 2013).

**Table 1:** mRNA and protein expression of balloon cells (BC) and giant cells (GC) of neuro-glial markers.

Category	Cell marker	BC	GC	Function	Notes and references
Neuronal stem cell marker	CD34	+	+	Stem cell marker	Positive in BC/GC in white matter (Fauser et al., 2004) Expressed in 7.5% of BC (Oh et al., 2008)
	CD133	+	+	Stem cell marker	BC (Ying et al., 2005) GC (Yasin et al., 2010)
	SOX2	+	+	Stem cell marker	BC (Yasin et al., 2010) GC (Yasin et al., 2010; Arceneaux et al., 2024)
	β1-integrin	+	+	Neural stem cell behavior modulator	BC/GC (Yasin et al., 2010)
	Nestin	+	+	Modulator of differentiation and migration of neural stem cells	57.1% of cells in BC (Marin-Valencia et al., 2014; Oh et al., 2008; Orlova et al., 2010) 80% of cells in GC (Crino et al., 1996; Mizuguchi et al., 2002)
	Vimentin	+	+	Radial glia marker and Neuronal stem cell marker	53.1%; 63.1% of cells in BC (Urbach et al., 2002; Oh et al., 2008; Marin-Valencia et al., 2014) GC (Hirose et al., 1995; Mizuguchi et al., 2002; Arceneaux et al., 2024)
	FGF2	+	+	Regulates differentiation in neuronal stem cells	BC/GC (Ueda et al., 2011)
Neuronal progenitor marker	MAP1B	+	+	Modulates neuronal migration and dendritic outgrowth	BC (Crino et al., 1997; Marin-Valencia et al., 2014); GC (Yamanouchi et al., 1997)
	Doublecortin	+	+	Neuronal progenitor cell marker	Expressed in a smaller number of cells in BC vs. GC (Mizuguchi et al., 2002), GC (Lee et al., 2003)
	FGF13	++	++	Modulates neuronal differentiation	BC (Wu et al., 2021a), mRNA expression is also shown in GC (Wu et al., 2021b).
	GFAP-*δ*	+	+	GFAP isomer in neuronal stem cells	BC/GC (Martinian et al., 2009)
Mature neuronal marker	α-internexin	+	+	Maintains dendritic structure	BC (Marin-Valencia et al., 2014) Expression 50–70%; mRNA expression is also shown in GC (Crino et al., 1996)
	MAP2	+	+	Influences microtubule dynamics in neurons	BC (Oh et al., 2008) GC (Crino et al., 1996)
	NeuN	+	+	Neuronal marker expressed in cell nuclei	BC (Marin-Valencia et al., 2014) GC Heterogeneous expression (Lee et al., 2003)
	NSE (vs. GFAP)	+	+	Neuronal marker	NSE more than GFAP in BC vs. GC compared to GFAP (Jozwiak et al., 2006)
Mature glial marker	GFAP	+	+	Astrocytic marker	BC Expression heterogeneous (Urbach et al., 2002) 67% of BC (Oh et al., 2008) GC (Hirose et al., 1995; Sharma et al., 2004; Arceneaux et al., 2024)
	S100 *β*	n	+	Glial marker	GC (Hirose et al., 1995; Sharma et al., 2004)
	Cx43	+	n	Glial gap junction protein	BC (Garbelli et al., 2011)

n: “no study found” -: “not expressed” u: “Underexpressed”; +: Expressed (may be weakly expressed or normal expression); ++: “Overexpressed”; +++: “Strongly Expressed.”All studies examined human cortical samples. Unless otherwise noted, all studies are immunohistochemical studies of protein expression.

Neuronal-glial markers expressed in BC/GC can be separated by their stage of differentiation: neuronal stem cell, neuronal progenitor, mature neuronal, and mature glial markers (Table 1). BC/GC commonly accumulate intermediate filaments vimentin and nestin, known to function in neuronal migration and differentiation in weeks 20–30 of embryonic development (Garbelli et al., 1999; Mizuguchi et al., 2002; Urbach et al., 2002; Oh et al., 2008). General markers of cellular immaturity, SOX2, OCT4, c-myc, KLF4, FOXG, were identified in more than 75% of BC examined (Orlova et al., 2010). The neuronal progenitor cell may express MAP1B, Doublecortin, FGF-13, or GFAP-*δ* variant, all present in BC/GC (Crino et al., 1997; Yamanouchi et al., 1997; Mizuguchi et al., 2002; Lee et al., 2003; Martinian et al., 2009; Marin-Valencia et al., 2014; Wu et al., 2021a; Wu et al., 2021b). Finally, markers of mature neuronal (*α*-internexin, MAP2, NeuN, NSE) and glial (GFAP, S100 *β*, Cx43) lineage are positive in BC/GC (Hirose et al., 1995; Crino et al., 1996; Urbach et al., 2002; Sharma et al., 2004; Jozwiak et al., 2005; Blandini et al., 2008; Garbelli et al., 2011; Marin-Valencia et al., 2014). Notably, they have variable GFAP and neurofilament staining patterns. In rare examples, co-expression of both markers was reported suggesting glial and neuronal lineage determination, that is, intermediate cells (Englund et al., 2005; Talos et al., 2008), while another study suggested that BC/GC have a stronger neuronal heritage (Mizuguchi et al., 2002). In agreement, single-cell analysis in tubers suggested that GC are of neuronal lineage despite the persistence of embryonic markers, such as nestin (Crino et al., 1996). As stated above, these discrepancies could be due to cell heterogeneity in FCDIIb and TSC samples.

Various neuronal-glial markers positive in BC/GC have been linked to epileptic activity and histological disturbances of cortical tissue in both FCDIIb and TSC. Doublecortin, an immature neuronal marker, is primarily found in migrating cells of the fetal central nervous system and correlates well with the degree of histological abnormality of the lesion (Mizuguchi et al., 2002). Fibroblast growth factor 13 (FGF13) plays a role in the differentiation of neurons during early embryonic development and is correlated with seizure frequency in FCDIIb and TSC cases (Wu et al., 2021a). Fibroblast growth factor 2 (FGF2) upregulation in BC has been shown to positively correlate with disturbed gliogenesis and neuroblast migration (Ueda et al., 2011). A survey of FGF2 across multiple MCD showed that the percentage of FGF2-IR can reflect the timing of insult in each cortical development disorder (Sugiura et al., 2008).

Interestingly, a recent customized machine-learning workflow trained to identify BC in tissue sections using a histological stain compatible with high-dimensional cytometry (BAIDEN), reported that BC express proteins associated with progenitor-cell identity (e.g., vimentin, SOX2, CD133, and EGFR) rather than mature-cell identity (e.g., β-III-tubulin, SMI-311, GFAP, and EAAT1), which tended to be decreased (Arceneaux et al., 2024).

### 4.2 mTOR pathway markers

MTOR is a tumor suppressor gene which codes a protein product (mTOR) pivotal to GC pathology because functional hamartin (TSC1) and tuberin (TSC2) inactivate the mTOR pathway. The protein kinase regulates, among others, cellular growth, proliferation and differentiation, autophagy, and immune responses (Panwar et al., 2023). In line with demonstrated parallels between BC and GC, IHC studies found a loss of tuberin and hamartin expression, as well as strong immunoreactivity for mTOR in BC (Jozwiak et al., 2004; Grajkowska et al., 2008; Table 2). Considering the large size of BC/GC, numerous studies have also looked for evidence of increased mTOR downstream markers in BC/GC. IHC studies have shown increased expression of pS6K1, pAkt, pPDK, and p4EBP1 in both BC/GC (Baybis et al., 2004; Schick et al., 2006; Schick et al., 2007a; Rossini et al., 2017). However, in a study designed to assess whether the PI3K pathway, a modifier of the mTOR pathway, differentiates BC from GC, it was found that, when compared to GC, BC showed elevated upstream modifiers of TSC1 and TSC2, p-PDK1 and p-Akt, but similar levels of TSC1/TSC2 markers downstream the pS6 marker, suggesting recruitment of different factors in the molecular pathogenesis of GC in cortical tubers vs. BC in FCDIIb (Schick et al., 2007a). The presence of elevated upstream markers in BC that is absent in GC may suggest that the same therapy would not be effective in FCDIIb, but this has not been demonstrated yet.

**Table 2:** mRNA and protein expression of balloon cells (BC) and giant cells (GC) of mTOR and cell growth markers.

Category	Cell marker	BC	GC	Function	Notes and references
	pAkt	++	+	TSC 1 and 2 suppressor	mRNA data suggest normal expression in GC but increased in BC (Rossini et al., 2017; Schick et al., 2007a)
mTOR pathway Cell growth marker	Hamartin	u	-	Tumor suppressor; mTOR suppressor	BC/GC (Jozwiak et al., 2004; Grajkowska et al., 2008)
	Tuberin	u	-		
	pPDK	++	++	Signal transduction in mTOR	mRNA expression is also shown in BC/GC (Rossini et al., 2017; Schick et al., 2007a)
	p4EBP1	+	+	Translation initiation factor	mRNA expression is also shown in BC/GC; More focal, more variable expression in BC (Yasin et al., 2010)
	pTuberin	++	++	Inactivated tuberin/TSC2	Only shown in mRNA so far (Schick et al., 2007a)
	Actin Stress Fiber	++	++	Disrupts actin skeleton	Only shown in mRNA so far (Schick et al., 2007a)
	pS6	++	++	Protein synthesis, cell proliferation, apoptosis, and metabolism	BC/GC (Rossini et al., 2017)
	p-p70(S6k)	++	++	Cell proliferation, growth, and cell cycle progression	Only shown in mRNA so far (Schick et al., 2007a)
Cell growth pathways	MCM2	+	+	G1 cell stage marker	unable to be activated with stem cell mitogens in BC/GC (Yasin et al., 2010)
	IGF2	++	+	Insulin growth factor	Only shown in mRNA so far; Normal expression in GC (Baybis et al., 2004)
	Ki67	-	+	Cell proliferation marker and immature neuronal marker	BC (Munakata et al., 2013) GC (Crino et al., 1996; Crino et al., 1997)
Inflammation markers	LILRB2	+	+++	Modulates neurite growth, synaptic plasticity, and inflammatory responses	BC/GC (Yue et al., 2019)
	TLR4	++	++	Toll-like receptor involved in neurogenesis	BC/GC (Arena et al., 2019)
	IL-1β	++	++	Proinflammatory cytokine involved in neuronal development	BC/GC (Arena et al., 2019)
	IL-6/IL-6R	+++	+++	Proinflammatory cytokine involved in neuronal development	Colocalization with GFAP in BC, but not GC (Shu et al., 2010)
	IL-17/IL17R	+	+	Activates innate and adaptive immunity	2x higher compared to cortex in BC/GC (He et al., 2013)
	TGF-β	u	u	Regulates response to cell injury, including modulating cell growth	Only shown in mRNA so far (Baybis et al., 2004)
	FPR2	+	+	Inflammatory resolution in CNS; Negatively correlated with NF-kB	Weakly shown in GC/BC (Huang et al., 2022)

-: “not expressed” u: “Under expressed”; +: Expressed (may be weakly expressed or normal expression); ++: “Overexpressed”; +++: “Strongly Expressed.”All studies examined human cortical samples. Unless otherwise noted, all studies are immunohistochemical studies of protein expression.

Since the mTOR pathway is involved in a complex network of other protein pathways, Baybis et al. furthered their investigation by delineating transcription profiles of BC/GC in related cell growth factors. According to the study, similar expression changes in BCs and GCs included reduction of c-fos, hairy enhancer of split–1, and TGF-*β*1 and TGF-β2. However, GC demonstrated increased expression of AP-1, c-ret, ICAM-1, NF-kB, TGFR2, and VEGF, and reduced expression of c-jun, CaMKII, Erb, and platelet-derived growth factor receptor in comparison to BCs. The authors also demonstrated increased IGF2 in BC compared to GC based on single-cell mRNA data (Baybis et al., 2004).

### 4.3 Immune system markers

Various markers positive in BC/GC reflect alterations in immune activity (Table 2). Interleukins 6 and 17 (IL-6, IL-17) and their receptors are involved in the inflammatory response and demonstrated to be elevated in BC/GC. Notably, IL-6 is also involved in neuronal development and seen to be colocalized with GFAP in BC but not GC (Shu et al., 2010; He et al., 2013). Another cytokine, IL-1β, along with TLR4, also are highly expressed in BC/GC (Arena et al., 2019). Leukocyte immunoglobulin-like receptor subfamily B member 2 (LILRB2) is a neuronal progenitor cell marker, as well as a marker of inflammatory responses. It is involved in neurite growth, synaptic plasticity, and inflammatory responses. A study demonstrated that increased protein concentration of LILRB2 in BC/GC has a negative correlation with seizure frequency (Yue et al., 2019). Increased levels of Formyl Peptide Receptor 2 (FPR2) in BC/GC is also negatively related with NF-kB and seizure frequency in TSC and FCDIIb (Huang et al., 2022). Other markers, such as TGF-β, which regulates cell response to injury, was demonstrated to be under-expressed in BC/GC mRNA by single-cell analysis (Baybis et al., 2004).

### 4.4 GluR and GABA_A_R subunit markers, and glutamate and chloride cation transporters

The composition of GABA_A_ and glutamate receptor (GABA_A_R and GluR) subunits evolves throughout neuronal/glial development. Studies have used this principle to understand the identity of abnormal FCDIIb and TSC cells (Table 3). FCDIIb lesions, defined by their BC composition, demonstrated high GABA_A_ receptor subunit α4:α1 ratio. Comparatively, FCDIIa lesions (which lack BC) had low GABA_A_R α4:α1 ratio, suggesting BC may modify or directly contribute to altered GABA_A_R subunit composition. Investigation of TSC lesions showed analogously increased GABA_A_R α4:α1 ratio, further supporting similarities of BC/GC identity (Talos et al., 2012). Reduced expression of GABA_A_R α1 is linked to reduced sensitivity to benzodiazepines and barbiturates, which are GABA_A_R modulators indicated for select types of seizures, in these pathologies. Comparison of glutamate receptor expression, including AMPAR and NMDAR, have also been measured across BC/GC cells. Increases in GluA1 and GluA4 subunits are consistent with an immature GluR expression profile (Blandini et al., 2008; Talos et al., 2008). Although BC/GC express GluR and GABA_A_R subunits, no studies have demonstrated the ability to assemble into functional receptors.

**Table 3:** mRNA and protein expression of balloon cells (BC) and giant cells (GC) of glutamate and GABA subunit markers.

Category	Cell marker	BC	GC	Function	Notes and references
Glutamate receptor subunit	GluA1	++	+	Subunit of AMPAR; synaptic plasticity	BC (Oh et al., 2008); Weakly expressed in GC (Talos et al., 2008)
	GluA2	+	u	Subunit of AMPAR; synaptic plasticity	Normal expression in BC (Oh et al., 2008); GC (Talos et al., 2008)
	GluA3	++	u	Subunit of AMPAR	BC (Oh et al., 2008); GC (Talos et al., 2008)
	GluA4	+	++	Subunit of AMPAR; synaptic plasticity	Normal expression in BC (Oh et al., 2008); GC (Talos et al., 2008)
	GluN1	+++	+	Subunit of NMDAR	BC (Oh et al., 2008); Normal expression in GC (Talos et al., 2008)
	GluN2A/B	+++	+	Subunit of NMDAR	BC (Oh et al., 2008); Weak expression in GC (Talos et al., 2008)
GABA receptor subunit	GABA_A_R α1	u	u	Subunit of GABA_A_R; mature neuronal cell marker	BC/GC (Talos et al., 2012; White et al., 2001)
	GABA_A_R *α*4	+	+	Subunit of GABA_A_R; immature neuronal cell marker	Normal expression in BC/GC (Talos et al., 2012)
Transport protein	KCC2	u	u	Neuron-specific chloride potassium symporter	BC/GC (Talos et al., 2012)
	NKCC1	+	+	Na–K–Cl co-transporter in both neuron and glia	BC/GC (Talos et al., 2012)
	EAAT2	+	n	Glial marker; glutamate regulator	BC (Gonzalez-Martinez et al., 2011)
	EAAT3	u	+	Neuronal marker, glutamate regulator	BC (Gonzalez-Martinez et al., 2011) GC (White et al., 2001)

-: “not expressed” u: “Under expressed”; +: Expressed (may be weakly expressed or normal expression); ++: “Overexpressed”; +++: “Strongly Expressed.”All studies examined human cortical samples. Unless otherwise noted, all studies are immunohistochemical studies of protein expression.

The expression levels of protein transporters further elucidate BC/GC function (Table 3). Chloride cation transporters KCC2 and NKCC1 are downstream targets of mTOR and also the target of the drug bumetanide, respectively (Bakouh et al., 2024). Talos et al. demonstrated decreased KCC2 and increased NKCC1 levels in TSC and FCDIIb lesions. This expression profile is associated with increased neuronal excitability and enhanced seizure susceptibility (Ben-Ari, 2014). At the cellular level, high NKCC1 expression occurred in dysplastic neurons, as well as in GC and reactive astrocytes. In contrast, KCC2 was only expressed in dysplastic and normal-sized neurons, but not in undifferentiated GC or dysplastic astrocytes (Talos et al., 2012). The functional significance of increased NKCC1 in BC/GC is unknown. However, high expression of this chloride transporter could be a sign of immaturity (Rivera et al., 1999).

Glutamate homeostasis was also examined, as it is an important component of epileptogenicity regulated by glial cells. The glutamate transporter EAAT contains two isomers, each linked to a neuronal (EAAT3) versus a glial (EAAT2) lineage. In BC, there was higher EAAT2 than EAAT3 expression by IHC. Additionally, EAAT2 was associated with non-epileptic samples, while the EAAT3 stained heavily in epileptic sections. Together, the authors suggested BC may play a protective role in epileptogenesis (Gonzalez-Martinez et al., 2011). EAATs in GC have not been examined extensively. A mRNA study demonstrated increased abundance of EAAT3 in tuber slices compared to controls (White et al., 2001). After single-cell microdissection, the authors showed that EAAT3 mRNA was expressed in both dysmorphic neurons and GC, although its relative abundance was higher in dysmorphic neurons. Overall, these studies demonstrate that BC/GC are very similar in terms of gene/protein expression and that the differences are more quantitative than qualitative, reinforcing the idea of a common developmental origin.

## 5 Further insights into BC/GC identity from genetic studies

Single germline or somatic mutations in the mTOR pathway genes have emerged as a primary cause of FCDII (Pelorosso et al., 2019). While genetic mutations in TSC1 and TSC2 genes have been known for a long time (European Chromosome 16 Tuberous Sclerosis Consortium, 1993; Martin et al., 2017), the contribution of genetic mutations in FCDII took much longer to be recognized [for a concise review see (Lee et al., 2022)]. In TSC, pathogenic variants for TSC1 and TSC2 genes include deletion, nonsense, and missense mutations, leading to a loss-of-function of hamartin and tuberin (Caban et al., 2017). In FCDIIb, the presence of somatic mutations was first hypothesized in a case of hemimegalencephaly (HME, a congenital MCD with similar histopathology as FCDIIb), and TSC (Salamon et al., 2006). A few years later, whole-exome sequencing in paired brain–blood samples from HME patients identified *de novo* somatic mutations in PIK3CA, AKT3 and MTOR genes in brain samples from one third of affected individuals, indicating aberrant activation of mTOR signaling (Lee et al., 2012). Exome sequencing combined with mass spectrometry analyses validated genetic findings and identified variations in mutation burden across different cortical areas. This led to the conclusion that HME is a genetic mosaic disease caused by gain-of-function in the PI3K-AKT3-mTOR signaling pathway. Soon thereafter, using similar techniques, somatic missense mutations in PIK3CA, AKT3, MTOR and other associated genes were observed in both FCDII and HME cases (Baek et al., 2015; D'Gama et al., 2015; Lim et al., 2015; Nakashima et al., 2015; Pelorosso et al., 2019; Gerasimenko et al., 2023; Lopez-Rivera et al., 2023). In another study including a large cohort of surgical cases presenting with HME and FCDIIa/b, somatic gain-of-function variants in MTOR and its activators (AKT3, PIK3CA, RHEB), as well as germline, somatic and two-hit loss-of-function variants in its repressors (DEPDC5, TSC1, TSC2) were corroborated. Importantly, in the present context, analysis of pools of laser-captured microdissected cells and whole-genome amplification demonstrated that dysmorphic neurons and BC carry those pathogenic variants (Baldassari et al., 2019). More recently, in a large multicenter international collaboration that recruited 283 individuals with FCD, HME and TSC that underwent surgical resections, whole-exome and targeted-amplicon sequencing, as well as single-nucleus RNA sequencing, identified 75 mutated genes through intensive profiling of somatic mutations. In addition, many MCD mutated genes were dysregulated in some specific cell types, particularly in the astrocyte and excitatory neuron lineages (Chung et al., 2023). In agreement, a study in a homogeneous population of FCDII cases using single-nucleus RNA sequencing in morphologically-identified cells, showed that dysmorphic neurons and BC are molecularly distinct, with glutamatergic neuron-like and astrocyte-like identities, respectively (Baldassari et al., 2024). Interestingly, the same study demonstrated that BC display stronger expression of genes coding for secreted proteins (e.g., MFAP4 and IGFBP7), which could affect neighboring cells in a paracrine manner. Finally, some rare neurons displayed an intermediate phenotype, with normal somatic size and mitochondrial numbers, but with aberrant accumulation of intermediate filaments. In terms of the timing of the mutation and the possible origin of BC, based on the fact that other cell types including interneurons and microglia are mutated, the authors suggested that the mutations occurred prior to the divergence into different cell lineages, i.e., at the time of gastrulation, during gestational weeks 2–3 (Baldassari et al., 2024).

## 6 Electrophysiological and morphological findings in *ex vivo* slices

To the best of our knowledge, our group at UCLA, was the first and, thus far, the only one to record, *in vitro*, the electrophysiological properties of abnormal non-neuronal cells, in particular BC/GC, in FCDIIb and TSC pediatric cases. Although the UCLA pediatric epilepsy program started in earnest around 1988, initial electrophysiological studies sampled neocortical cells from all children undergoing surgery, including a wide variety of pathological substrates (e.g., FCD, infarct, Rasmussen’s encephalitis, tumors, etc.) (Wuarin et al., 1990; Wuarin et al., 1992; Dudek et al., 1995). ECoG was used sparingly to select neocortical sample sites, and cells were recorded blindly in slice preparations and categorized, a posteriori, based on electrophysiological properties and fluorescent markers or biocytin labeling. Not surprisingly, the findings were disappointing in that the electrophysiological properties of the sampled neurons were generally normal, and BC/GC cells were not observed.

Our success at recording BC/GC was the result of a number of notable factors: A selection of a homogeneous population of patients presenting with FCD and TSC, increasing the likelihood of finding abnormal cells; an improved method of neocortical sampling previous (MRI, PET) and during surgical procedures (ECoG) to determine the greatest cortical abnormality; the short period of time elapsed between sample resection and slicing (under 10 min); the use of IR-DIC optics to selectively identify abnormal-looking cells before patching; and the confirmation of the pathological substrate by an expert pathologist. Other *ex vivo* studies using human tissue samples from FCD, including FCDIIb, and TSC cases did not report electrophysiological recordings from BC/GC (Calcagnotto et al., 2005; Wang et al., 2007; Lozovaya et al., 2014; Bakouh et al., 2024; Ribierre et al., 2024).

**Figure 2:**
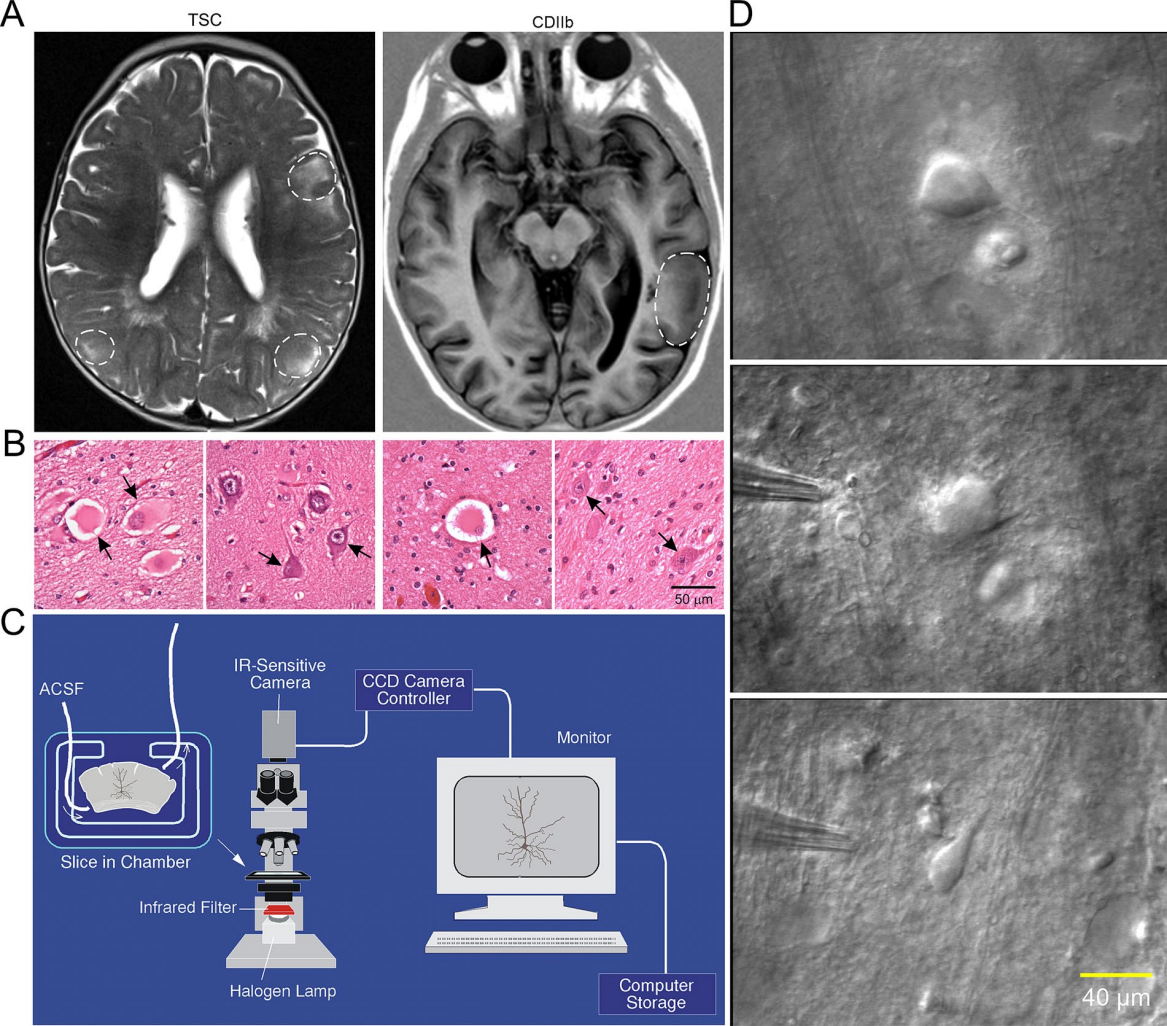
**(A)** Representative axial MRI scans of children (both 7 years old) with refractory epilepsy from TSC (left) and FCDIIb (right). There are multiple cortical tubers (dashed circles) in the patient with TSC and one focal area of cortical dysplasia in the left temporal lobe in the child with FCDIIb. **(B)** *Left panels*: Section of a tuber (originating from a 19-month-old male) showing abundant giant cells and disorganized collections of dysmorphic neurons. Arrows in left panel indicate giant cells in which the nucleus shows coarse chromatin, a pattern often seen in astrocytes. *Right panels*: Representative fields from a resection (originating from a 9-year-old female) showing features of FCDIIb. Note a balloon cell (arrow, left panel) with glassy eosinophilic cytoplasm, and dysmorphic neurons (arrows, right panel). The balloon cell shows “retraction” of cytoplasm from the neuropil (images are from sections stained with H&E). Scale bar represents 50 μm and applies to all panels. **(C)** Setup used for electrophysiological recordings from visualized cells in *ex vivo* brain tissue slices. An upright microscope equipped with differential interference contrast (DIC, Nomrarski) optics and infrared (IR) illumination allows visualization of individual cells. Slices are maintained in a custom-made chamber with oxygenated artificial cerebrospinal fluid (ACSF). IR images are stored in a computer. After electrophysiological recordings the slices are fixed in paraformaldehyde and processed for biocytin staining. **(D)** First ever IR-DIC images (top and middle panels) of BC/GC from a TSC patient (3.27 years). Notice the large size of BC/GC compared to a normal pyramidal neuron (bottom panel). Adapted from Cepeda et al. (2003, 2012). This and other figures use material previously published. Permission to use this material was obtained from Wiley and Elsevier Publishers.

Methods to visualize BC/GC in *ex vivo* slices: With the advent of IR-DIC microscopy (Dodt and Zieglgansberger, 1990), visualization of single cells in slices became feasible (Figure 2). This technique combines infrared light, which penetrates deeper in brain tissue, and Nomarski optics, which greatly enhances the contrast in unstained samples. One drawback of this technique when using brain slices is that, with age and increased myelination, it becomes more difficult to visualize individual cells. We were very fortunate in that our cohort comprised children as young as 2 months of age, up to 14 yr. Before 5 yr. of age, visualization is optimal, but BC/GC could be recorded even in the older cases. In addition, the younger the patient, the better the tissue preservation, the more resistance to hypoxia, and to the trauma caused by the slicing procedure. Brain slices could be maintained in good condition for up to 24 h.

**Figure 3:**
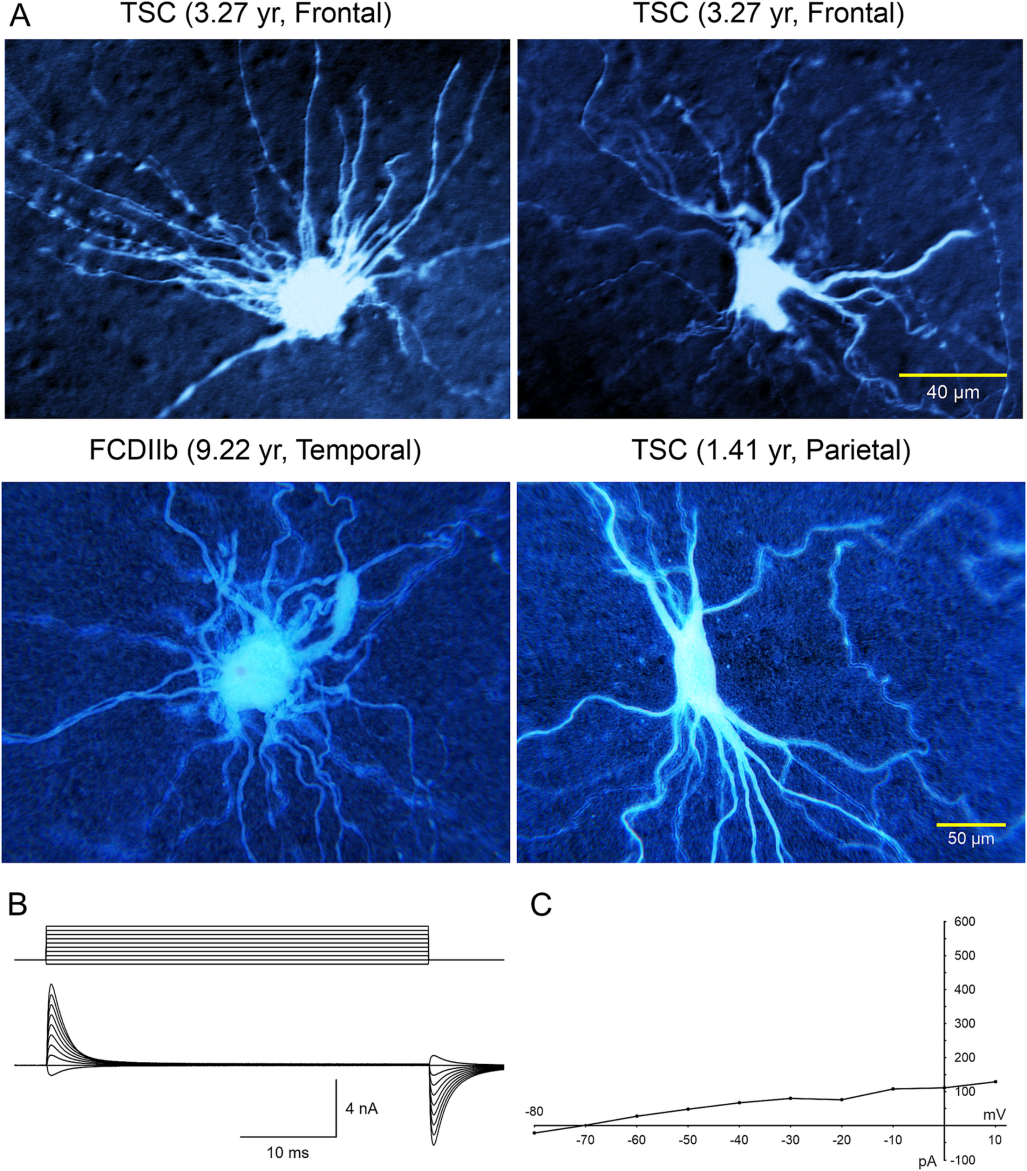
**(A)** Large cells recorded electrophysiologically in tissue slices from TSC and FCDIIb cases. Cells were recorded and subsequently stained with biocytin. Top left cell corresponds to the IR image in Figure 1 (top panel). The voluminous cell in the FCDIIb case had a very thick appendage and presented with an incipient Na^+^ current at depolarized potentials, while the other cells displayed no inward currents and only small outward currents. **(B)** Whole-cell patch clamp recording of the cell in top left. In voltage clamp mode, a series of hyperpolarizing and depolarizing voltage commands (from −80 to +10 mV) evoked practically no inward current and very small outward currents. The high input resistance suggested a neuronal phenotype; however this was dismissed as no Na^+^ or Ca^2+^ currents were observed. The patch pipette contained Cs-methanesulfonate-based internal solution. **(C)** Current–voltage plot of responses evoked by hyperpolarizing and depolarizing voltage commands in **(B)**. Only very small outward current could be measured. Calibration bar on the right also applies to left panels. The original biocytin images were modified for pseudo-color and brightness/contrast enhancement using Adobe Photoshop. Adapted from Mathern et al. (2000) and Cepeda et al. (2003, 2012).

Electrophysiological properties of BC/GC: The first IR-DIC images of GC (Figure 2D) from a TSC patient (3.27 yr. old) were obtained in 1997. In this case, GC were subsequently recorded using the whole-cell patch clamp technique in voltage clamp mode (Figures 3A). After breaking the gigaohm seal and applying a series of depolarizing step voltage commands, we noticed that some GC had an usually high membrane input resistance, suggesting a neuronal phenotype typically seen in immature neurons. Unexpectedly, inward (Na^+^ and Ca^2+^) or outward (delayed rectifier) currents were, respectively, absent or minimal (Figures 3B,C), suggesting the possible existence of “silent” neurons. After processing the slices for biocytin to obtain a more detailed picture of those cells, we noticed their bizarre morphology consisting of abundant undulating processes stemming from round or oval somata, the absence of dendritic spines and instead the presence of varicosities, and, more importantly, a definite axon could not be found. We initially called these cells “atypical.” IHC staining of tissue from the same case, demonstrated that many of these “atypical” cells were positive for NeuN. However, a few cells were labeled by both NeuN and GFAP, suggesting a glial or mixed phenotype (Mathern et al., 2000). In fact, the morphology appeared consistent with that of fibrillary astrocytes. As more cases of FCDIIb and TSC were examined both electrophysiologically and morphologically, we came to the realization that these “atypical” cells were indeed the “grotesque,” “bizarre,” “monstrous,” BC/GC described in classic anatomical studies (Cepeda et al., 2003). But while BC/GC have attracted most of the attention, we have to emphasize that medium- and small-sized, variably shaped cells also abound in FCDIIb and TSC tissue. Many of these could correspond to the “stellate” or “spider” cells reported by pioneer investigators of TSC cases.

**Figure 4:**
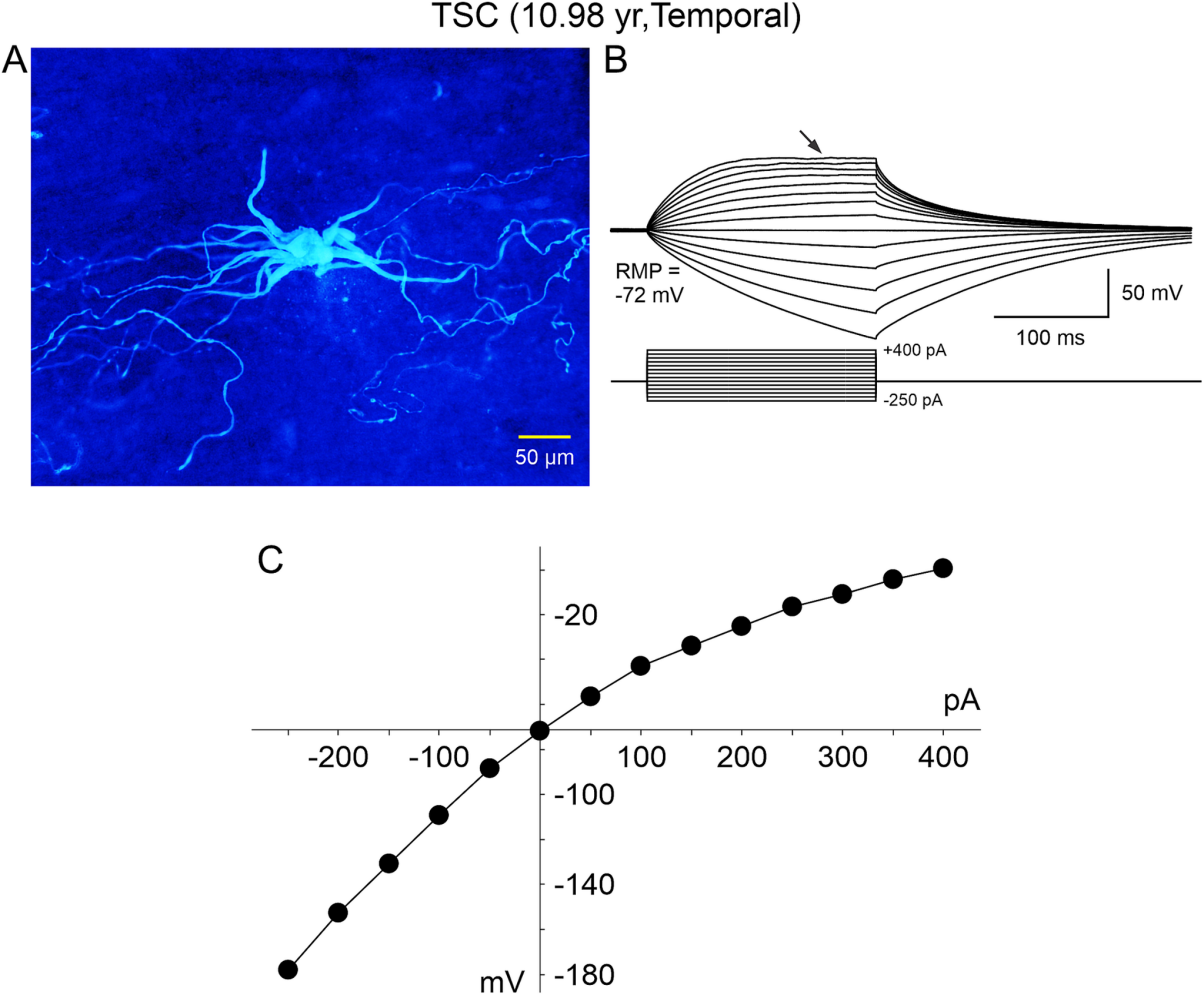
A “spider”-like cell recorded in a TSC case. **(A)** Biocytin-filled cell showed multiple thick processes emerging from an ill-defined, gnarled soma. As they distanced from the center, these processes tapered and became progressively thinner, swerving around haphazardly. The thin processes contained numerous varicosities, similar to those illustrated in Machado-Salas (1984) study (Figure 1) and speculated as being a point of neuronoglial interaction by a reactive astrocyte and a deteriorated cortical pyramidal neuron. **(B)** This cell was recorded in current clamp mode (K-gluconate as the pipette internal solution). A series of negative and positive step currents induced voltage deflections similar to those evoked in a “model” cell. However, no action potentials could be evoked even at very depolarized potentials and instead a strong rectification occurred (arrow). **(C)** Current–voltage plot of the changes in voltage evoked by increasing negative and positive current pulses. Notice that with negative current steps the current–voltage relationship is practically linear, whereas positive current steps induce strong rectification. Adapted from Cepeda et al. (2012).

**Figure 5:**
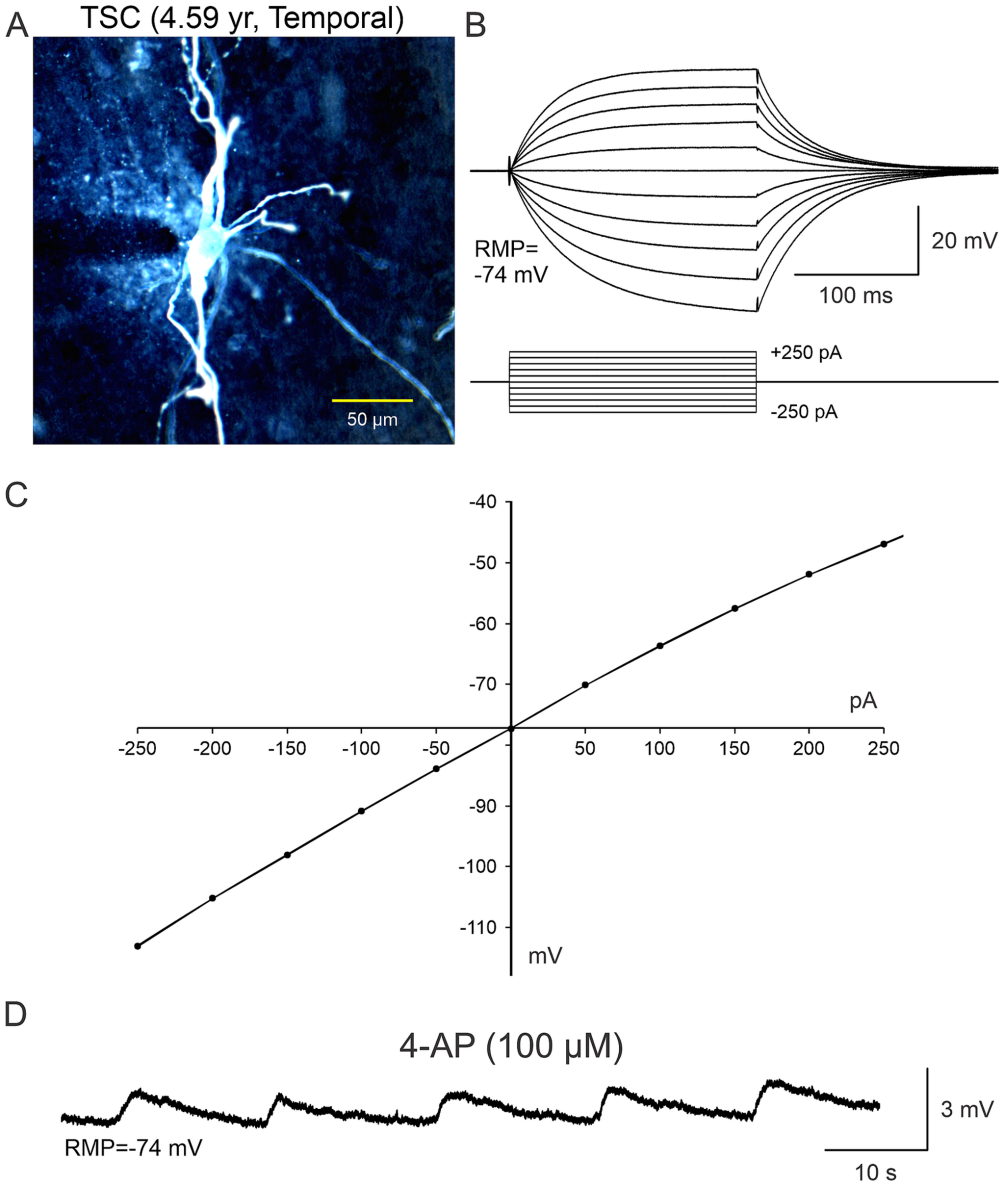
In terms of the functional role of non-neuronal cells **(A–C)**, in some electrophysiological recordings slices were bathed in ACSF solution containing 4-aminopyridine (4-AP), a K^+^ channel blocker that increases neurotransmitter release as well as K^+^ concentration in the extracellular milieu. It became evident that BC/GC were not completely isolated from the environment as they were capable of sensing changes in K^+^ concentration, that was manifested by small, rhythmic membrane oscillations **(D)**. Adapted from Levinson et al. (2020).

The electrophysiological membrane properties of non-neuronal cells, including BC/GC in FCDIIb and TSC, were examined in more detail in subsequent studies by our group (Cepeda et al., 2005a, 2006, 2010). Passive membrane properties showed that most BC/GC had a large membrane capacitance, consistent with their large size, the input resistance was more variable, but it was generally high. In terms of active membrane properties, recordings in current clamp mode showed that most cells had a hyperpolarized resting membrane potential, and could not generate action potentials even at very depolarized voltages (Figures 4, 5B). Analysis of the current–voltage (IV) plots revealed that some cells had a clearly linear relationship, consistent with an astroglial phenotype, while others displayed rectification at hyperpolarized or depolarized membrane potentials, consistent with a neuronal phenotype. In addition, small outward currents usually could be appreciated, and very rarely an incipient inward current occurred (Cepeda et al., 2012).

**Figure 6:**
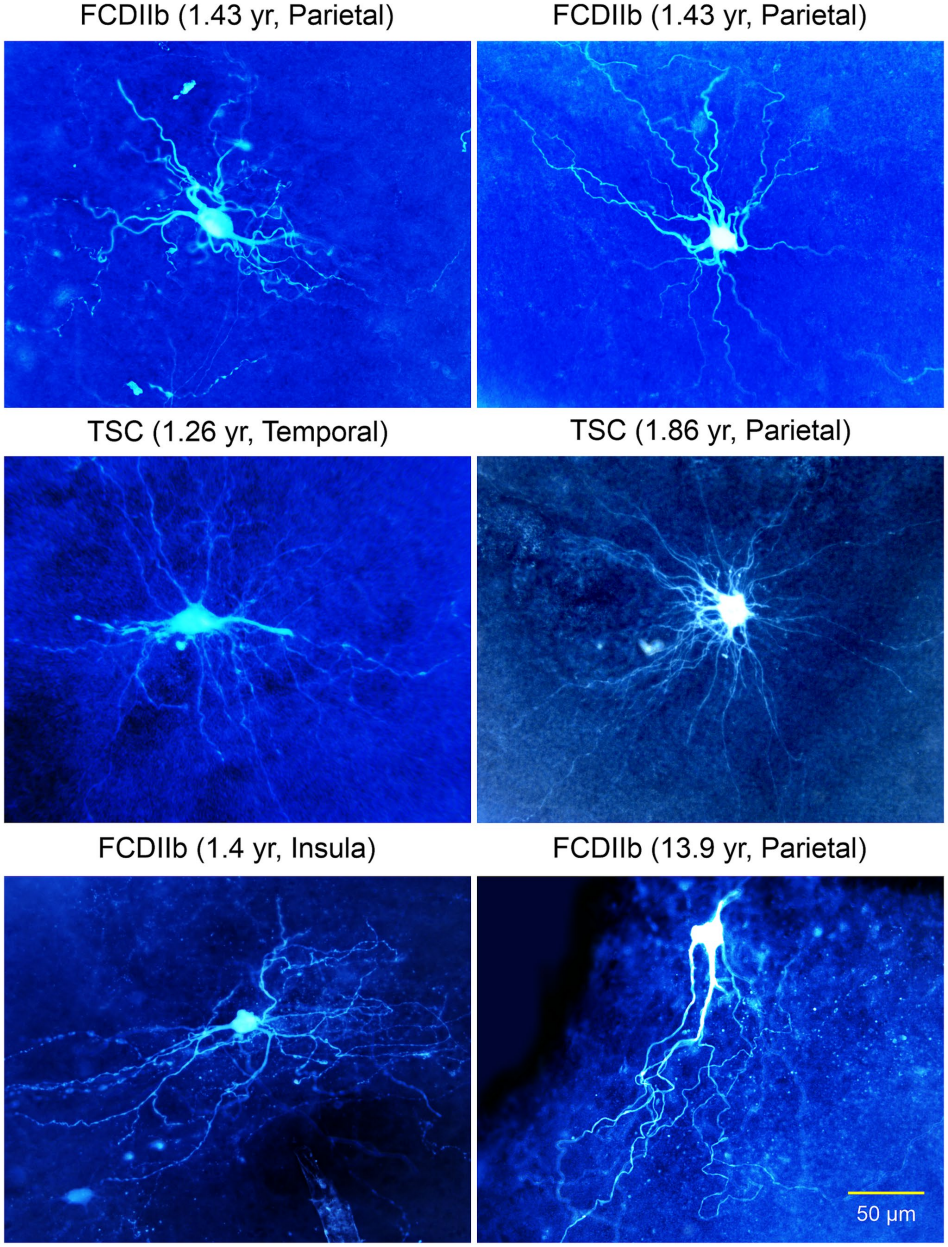
Gallery of non-neuronal cells with “neuronal”-like properties recorded in FCDIIb and TSC cases. Some commonalities included the inability to generate action potentials, lack of synaptic inputs, and absence of dendritic spines. The cell in the right middle panel appeared similar to a glial cell, however, its electrophysiological properties were more neuronal-like, e.g., high input resistance. Some of these cells appear similar to the “stellate” cells reported by Huttenlocher and Heydemann or by Ferrer et al. in 1984. Importantly, they do not have an axon. Adapted from Cepeda et al. (2003, 2005a,b, 2006).

**Figure 7:**
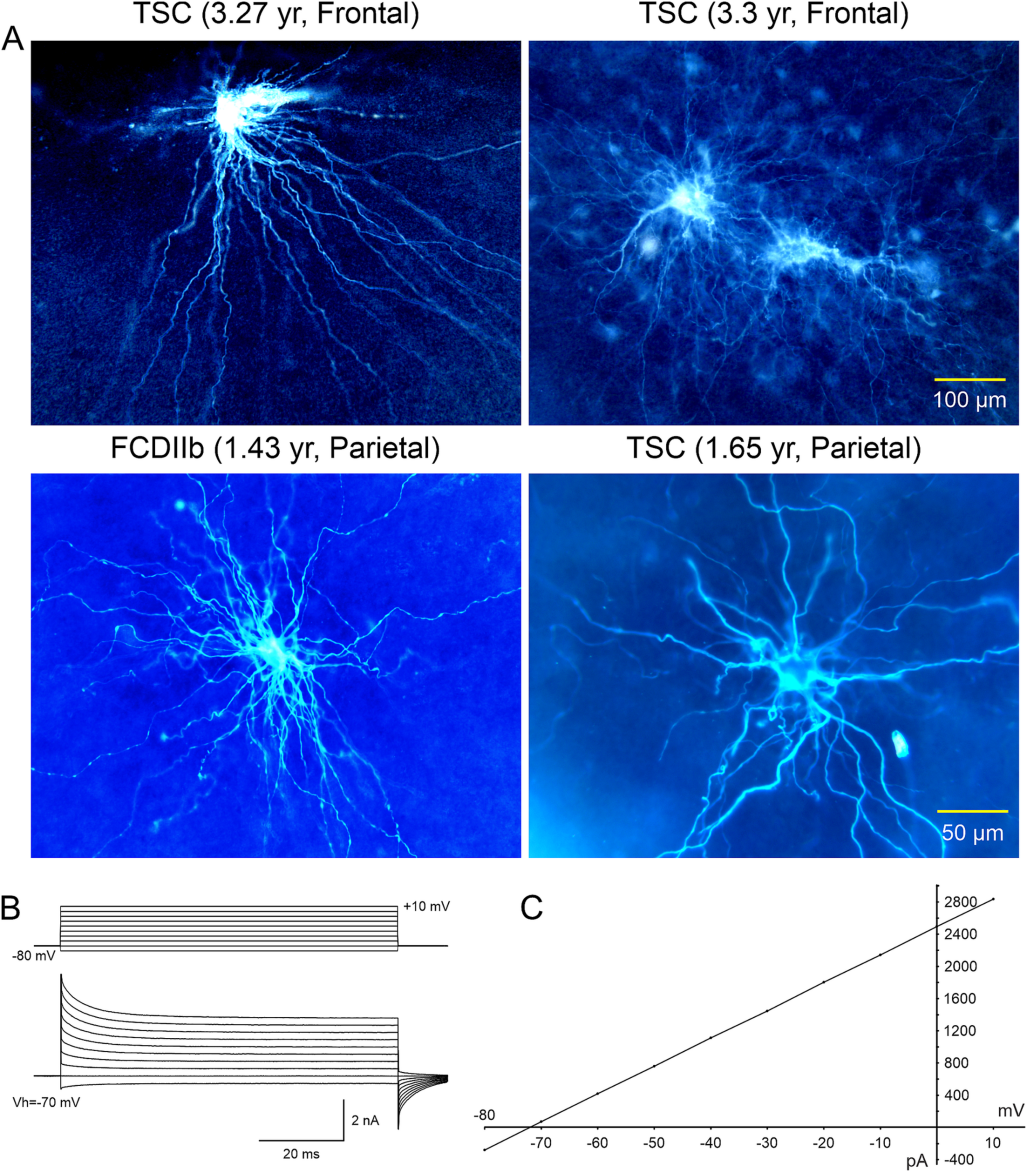
**(A)** Cells with glial-like properties recorded in TSC and CDIIb cases. The electrophysiology of depicted cells was more typical of those of astrocytes, except for the large size and extended space domain. The two giant cells in top right panel appear coupled via gap junctions. **(B)** One of the coupled cells (top right panel) was recorded in voltage clamp mode. It displayed a very low input resistance and large cell membrane capacitance. **(C)** The plot shows a typical linear current–voltage relationship as well as very large outward currents. Adapted from Cepeda et al. (2012).

**Figure 8:**
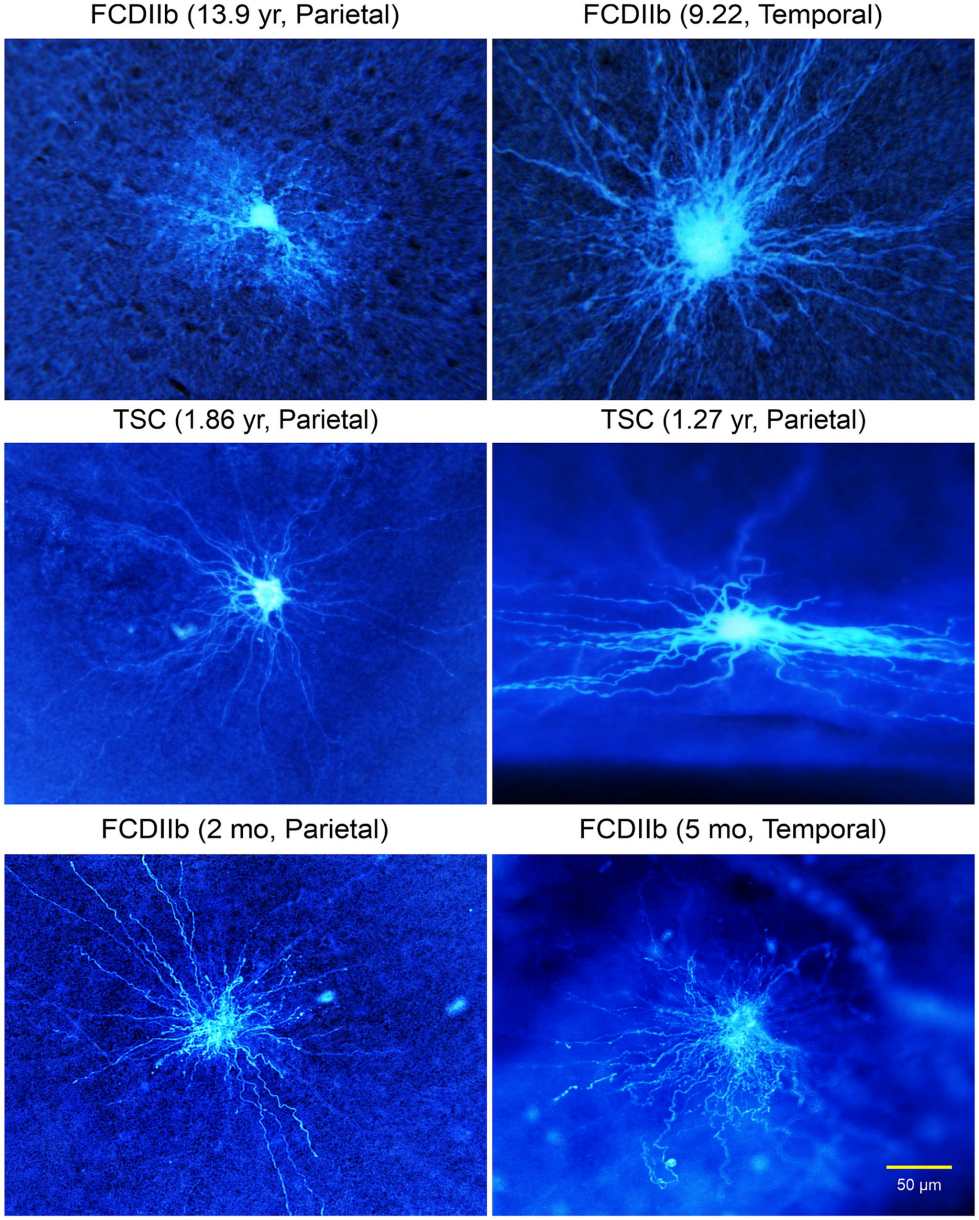
Gallery of typical glial cells. The astrocyte in top left panel looks more normal compared to the other cells, in particular the enlarged cell in the top right panel. All cells had abundant processes extending for several hundred microns. The cell in the right middle panel was recorded in the subpial area, hence the very different, flattened space domain of its processes. Adapted from Cepeda et al. (2012).

It soon became evident that a simple categorization of non-neuronal cells as just BC/GC in FCDIIb and TSC, could not account for the wide variety of morphological and electrophysiological features of these cells. Thus, while some of the non-neuronal cells appeared “neuronal-like” (Figure 6) and others appeared “glial-like” (Figures 7, 8), still others defied classification. Although calling non-neuronal cells “neuronal-like” seems paradoxical, it is not unprecedented. We recently recorded human neural stem cells implanted in a mouse model of Huntington’s disease. Some of the cells displaying immature membrane properties, including very high input resistance, did not fire action potentials when depolarized, and received no or rare synaptic inputs (Holley et al., 2023). If we assume that BC/GC are frozen in a progenitor neural stem cell stage, it is possible that some membrane properties appear neuronal-like, while others are not yet typical of neurons.

**Figure 9:**
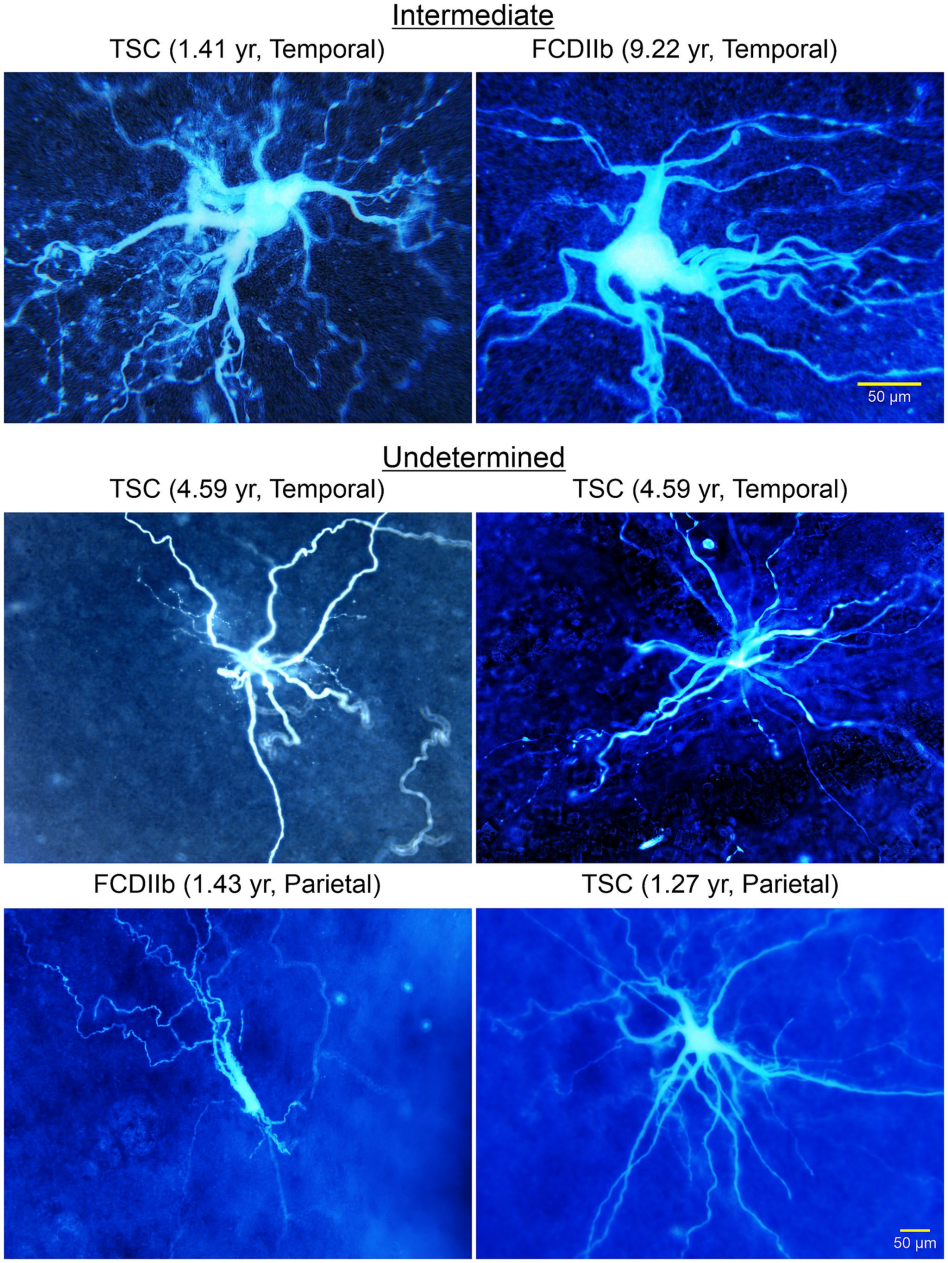
A wide variety of cells was observed in TSC and FCDIIb tissue samples. Some cells had a neuronal, even pyramidal-shaped soma (top right panel). However, the processes lacked spines and the thick (apical-looking) dendrites quickly metamorphosed into a multitude of fine branches. These cells are considered “intermediate” for their glioneuronal features. Other cells could not be determined (middle and bottom panels). Some appeared as just a few thick processes but seemingly lacking a defined soma. The cell in the bottom left panel appeared as a deteriorating cell similar to the aberrant cortical neurons observed by Machado-Salas (1984, Figure 2). Adapted from Cepeda et al. (2012).

**Figure 10:**
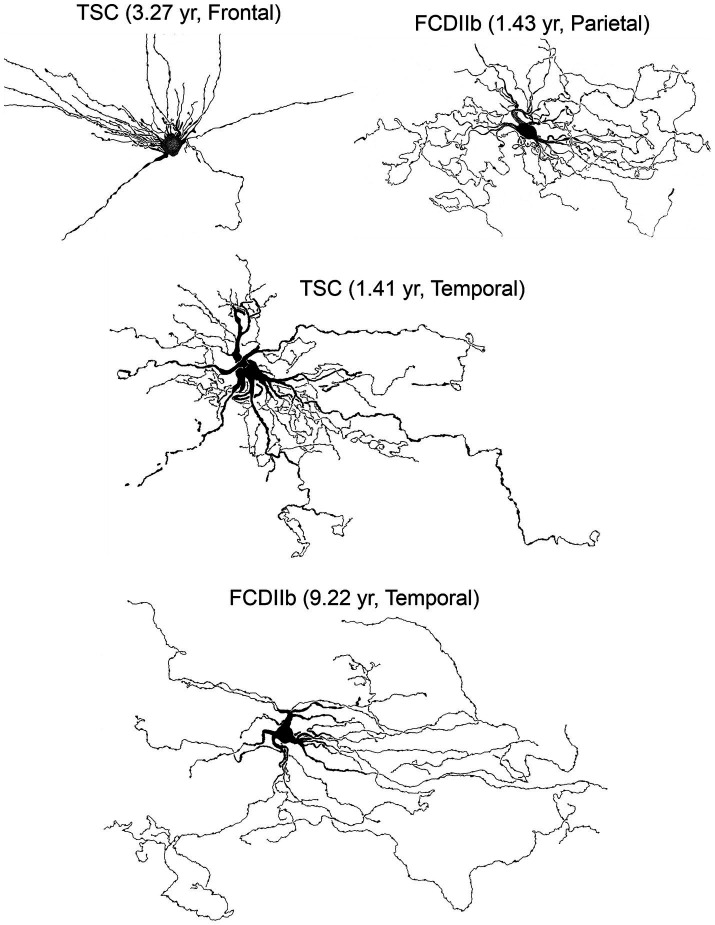
Some of the biocytin-filled cells shown in previous figures were used to construct camera lucida drawings (courtesy of the late Robin S. Fisher). Cells displayed various somatic shapes but all possessed long, waving processes with numerous varicosities. Adapted from Cepeda et al. (2003, 2006, 2012).

Other types of abnormal non-neuronal cells: We later described the existence of “intermediate cells” that appear to have a clear pyramidal-shaped soma and a nascent apical dendrite, but in more distal regions they display bushy, wavy processes, reminiscent of those observed in BC/GC (Figures 9, 10; Cepeda et al., 2012). Finally, after separating cells into “neuronal-like,” “glial-like,” and “intermediate,” still some cells could not be categorized, and we call them “undetermined” (Figure 9). Indeed, we also recorded cells that looked like dysmorphic or reactive astrocytes. This rich variety could reflect the fact that the degree of neuronal vs. glial marker expression varies from cell to cell, suggesting that these cells conform a heterogeneous group. This is consistent with observations in TSC histological samples showing a wide spectrum of abnormal cells including dysplastic neurons, giant neuroglial cells, giant and dysplastic astroglia, and reactive astrocytes (Talos et al., 2008). Indeed, based on the pediatric epilepsy material we have examined in the past 30 years we found that non-neuronal cells represent a heterogeneous group, with a wide variety of cell shapes, sizes, and dendritic elaboration, suggesting diverse function or lack thereof, as well as different roles in epileptogenesis.

In sum, before the introduction of IR-DIC microscopy, it was believed that BC/GC could play an active role in epileptogenesis, based on complex morphology and exuberant dendrites (Spreafico et al., 1998; Schwartzkroin and Walsh, 2000). A paradigm shift occurred when we demonstrated conclusively that BC/GC cells were incapable of generating action potentials, did not appear to receive synaptic inputs, and were unresponsive to iontophoretic application of excitatory aminoacids (Mathern et al., 2000; Cepeda et al., 2003). The absence of synaptic inputs is consistent with IHC studies by other groups showing that cortical tubers have reduced expression of synapsin I, as well as other synaptic markers (Lippa et al., 1993; Toering et al., 2009). Overall, our findings support the idea that non-neuronal cells in FCDIIb and TSC are a heterogenous group that can come in many flavors, similar to IHC findings in TSC cases (Talos et al., 2008). This heterogeneity of non-neuronal cells in FCDIIb and TSC, calls for a re-evaluation of cell classification in these pathologies.

## 7 Functional considerations regarding BC/GC and their role in epileptogenesis

BC are pathognomonic of FCDIIb, resemble GC found in TSC, and many of these cells are similar to fibrillary astrocytes. Indeed, some authors have suggested that TSC, and by extension FCDIIb, could be classified as a pathology of astrocytes, a group that includes, among others, subependymal tumors, SEGA, Alexander disease, and gemistocytic astrocytoma (Sosunov et al., 2008). For example, despite the name astrocytoma, the GC in SEGA also express neuronal markers (Jozwiak et al., 2005; Jozwiak et al., 2006). Similarly, in Alexander disease, a genetic disorder of astrocytes caused by a dominant gain-of-function mutation in the GFAP gene, enlarged dysmorphic and reactive astrocytes with characteristic Rosenthal fibers can be found (Brenner et al., 2001; Messing et al., 2012). They are believed to be a product of elevated GFAP, secondary to increased protein synthesis driven by the mTOR pathway. Of relevance, Rosenthal fibers also have been observed in some TSC and FCDIIb cases (Khanlou et al., 2009; Hirfanoglu and Gupta, 2010; Blumcke et al., 2021). Similar histopathologies in these disorders support the idea that FCDIIb and TSC could be considered, at least in part, a disease of astrocytes. This idea has been reinforced by recent single-cell genomic and transcriptomic profiling demonstrating that BC in FCDIIb are closely related to astrocytes, whereas dysmorphic neurons are more akin to glutamatergic neurons (Baldassari et al., 2024).

In normal and pathological conditions, astrocytes play a critical role in, among others, buffering K^+^ and glutamate, providing energy substrates, neurotransmitter precursors, purines, growth factors, and gliotransmitters (Sofroniew and Vinters, 2010). Importantly, astrocytes are an integral part of the tripartite synapse and modulate neuronal activity via feedback mechanisms. Aberrant synaptic communication between neurons and glia may thus contribute to neural pathologies (Liu et al., 2023). If some BC/GC can be considered enlarged astrocytes, what could be their significance in FCDIIb and TSC? Before attempting to answer this question, it is important to determine whether BC/GC establish direct contact with neurons and vice versa. In his landmark Golgi study, Machado-Salas noticed the existence of GC-neuron contacts, suggesting a possible substrate for interaction (Machado-Salas, 1984). Also, as noted earlier, an ultrastructural study reported the existence of rare neuroglial junctions (Trombley and Mirra, 1981). However, direct contacts between neurons and BC/GC seem to be an exception. More likely, the presence of BC/GC leads to aberrant synaptic organization. Because axon terminals slated to innervate cortical areas are now occupied by BC/GC unable to form functional synaptic connections, those areas could become hyperexcitable (Cepeda et al., 2003; Cepeda et al., 2006; Abdijadid et al., 2015). Interestingly, studies have demonstrated that areas with larger accumulation of BC do not appear to be epileptogenic, in fact they display less paroxysmal activity than adjacent areas (Boonyapisit et al., 2003). This agrees with FDG-PET characterization of FCDIIb and TSC lesions prior to surgical resections demonstrating that BC are localized to areas of hypometabolism (Luat et al., 2007; Wang et al., 2023), likely related to the inability of BC to generate action potentials (Cepeda et al., 2006).

Investigation of glutamate clearance showed BC outside of ictal onset areas had an increased expression of glial glutamate transporters (GLT1/EAAT2) (Gonzalez-Martinez et al., 2011). Increased neuronal glutamate transporters also were found in cortical tubers (White et al., 2001). Therefore, it is possible that BC/GC play a protective role in epileptogenesis, regulating neuronal synaptic transmission via the tripartite synapse. In support, we recently demonstrated that BC/GC can sense changes in neurotransmitter and K^+^ concentration and display slow rhythmic membrane oscillations during high levels of neuronal activation (Levinson et al., 2020; Figure 5). Similar to BC/GCs are intermediate cells, which display both neuronal and glial markers and do not generate action potentials (Talos et al., 2008; Blumcke et al., 2011; Cepeda et al., 2012). Thus, while BC/GC and intermediate cells do not appear to participate actively in epileptogenic processes, they could instead dampen epileptic activity.

A recent review article asked the question of whether BC/GC in FCDIIb, TSC, and HME are friends or foes (Liu et al., 2024). The authors are correct in that there is no simple answer and their role in these pathologies is more nuanced, which may be related to the extent to which BC/GC are more glial-like or neuronal-like. If BC can effectively dampen epileptic activity, one may think that they are friends. This would agree with the fact that epileptogenesis is associated with the density of dysmorphic cytomegalic neurons (Stephenson et al., 2021) and not with that of BC/GC. However, even though BC/GC lack an axon and classical chemical synapses, gliotransmission is still possible. For example, a study designed to examine neuron–glia interactions in cell cultures and organoids from TSC and control cases demonstrated that TSC astrocytes displayed increased proliferation and changes in gene expression consistent with an enrichment of secreted and transmembrane proteins related to EGFR signaling (Dooves et al., 2021). Surprisingly, in neurons cultured with astrocyte-conditioned medium there was an increase in the percentage of GABA synapses (VGAT^+^) relative to glutamate synapses (VGLUT^+^), leading to altered excitatory-inhibitory balance. Considering that KCC2 is reduced in cortical tubers and FCDIIb lesions (Talos et al., 2012), GABA gliotransmitter release from BC/GC could become excitatory. Interestingly, studies have shown increased GABA levels along with reduced benzodiazepine receptors in brain tissue from TSC patients (Mori et al., 2012). Thus, GABA gliotransmission, if confirmed, could exacerbate the intrinsic epileptogenicity of dysmorphic cytomegalic pyramidal neurons (Cepeda et al., 2005b; Cepeda et al., 2007; Abdijadid et al., 2015). In addition, considering that BC/GC do not appear to participate in integrative functions associated with typical neurons, their energy demands could represent a metabolic burden for local neuronal circuits.

## 8 Therapeutic implications

Without knowing with certainty whether BC/GC and other non-neuronal cells in FCDIIb and TSC are protective or deleterious, targeting these cells specifically or the mTOR pathway in general, the possible outcomes are difficult to predict. Because of the diffuse nature of these changes affecting not only the lesion or tuber areas but also the perilesional/perituberal regions, and the fact that they occur so early in brain development, it is unlikely that pharmacological manipulations will have a major impact on seizure-generation unless the lesions can be identified in utero and treatment starts immediately. A promising venue is CRISPR-based gene editing. For example, gene activation via targeting enhancers of haploinsufficient genes has been proposed as a therapeutic intervention in autism, as well other neurodevelopmental disorders (Chen et al., 2024). However, some symptoms in FCDIIb and TSC patients can be ameliorated using mTOR targeting drugs. For example, mTOR inhibitors everolimus and rapamycin have demonstrated the ability to decrease seizure activity in TSC human trials (Muncy et al., 2009; Krueger et al., 2013). Sirolimus, generic for rapamycin, also was shown to reduce the frequency of epileptic seizures in FCDII patients (Kato et al., 2022). However, no improvement was noted in another case (Hadouiri et al., 2020). A different clinical study in TSC and FCD patients showed that short-term everolimus had no adverse effects and there was a trend for lower pS6 (Leitner et al., 2022). In *ex vivo* slices, we demonstrated antiseizure effects of everolimus and these effects were more pronounced in TSC and FCD cases compared to non-mTOR-mediated pathologies (Cepeda et al., 2018). Another pharmacological target has been finding ways to reverse the depolarizing actions of GABA caused by high levels of NKCC1 and low levels of KCC2 (Talos et al., 2012), which facilitate seizure occurrence (Dzhala et al., 2005). A number of studies have demonstrated efficacy of bumetanide in the treatment of seizures and some symptoms of autism (Ben-Ari, 2017; van Andel et al., 2020; Soul et al., 2021; Ben-Ari and Cherubini, 2022). Other studies also indicate that the expression of innate immune markers is elevated in areas with high concentrations of BC in FCDIIb, particularly in the white matter (Zimmer et al., 2021). Thus, therapies targeting inflammation could be beneficial in FCDIIb. Similarly, a recent study reported signatures of cellular senescence (e.g., p53/p16 expression and senescence-associated *β*-galactosidase activity) in dysmorphic neurons and BC found in FCDIIb cases, suggesting that senolytic drugs such as dasatinib and quercetincan can be used to reduce seizure activity (Ribierre et al., 2024).

## 9 Conclusion

Any attempt to classify BC/GC into further subcategories cannot be based on purely morphological or electrophysiological grounds. Only by integrating electrophysiological observations, morphology, protein and gene (mRNA) expression, can we determine with more accuracy cell type and potential function. Morphology alone can be misleading (e.g., BC/GC being epileptogenic or “stellate” neurons lacking an axon). By definition, neurons have the capacity to generate an action potential, they possess an axon able to transmit electric signals, integrate synaptic inputs, and release neurotransmitters and/or neuromodulators. We can conclude that BC/GC are a subtype of non-neuronal cells and are functionally similar to astrocytes. However, some BC/GC also display some neuronal properties typically found in immature neural stem cells. Thus, although definitely not neuronal, BC/GC, as well as some medium-sized cells unable to fire action potentials, can be categorized into different subgroups based on morphological and electrophysiological properties as neuronal-like or glial-like. We hope that decontextualizing these cells from their pathology may elucidate the role of BC/GC in the pathophysiology of pediatric epileptic seizures caused by FCDIIb and TSC.
